# Statistical modeling to quantify the uncertainty of FoldX-predicted protein folding and binding stability

**DOI:** 10.1186/s12859-023-05537-0

**Published:** 2023-11-12

**Authors:** Yesol Sapozhnikov, Jagdish Suresh Patel, F. Marty Ytreberg, Craig R. Miller

**Affiliations:** 1 Program in Bioinformatics and Computational Biology, University of Idaho, Moscow, ID 83844 USA; 2 Department of Biological Sciences, University of Idaho, Moscow, ID 83844 USA; 3 Department of Physics, University of Idaho, Moscow, ID 83844 USA; 4 Department of Chemical and Biological Engineering, University of Idaho, Moscow, ID 83844 USA; 5 Institute for Modeling Collaboration and Innovation, University of Idaho, Moscow, ID 83844 USA

**Keywords:** Protein stability, Protein mutations, Stability prediction, Error prediction, Statistical model

## Abstract

**Background:**

Computational methods of predicting protein stability changes upon missense mutations are invaluable tools in high-throughput studies involving a large number of protein variants. However, they are limited by a wide variation in accuracy and difficulty of assessing prediction uncertainty. Using a popular computational tool, FoldX, we develop a statistical framework that quantifies the uncertainty of predicted changes in protein stability.

**Results:**

We show that multiple linear regression models can be used to quantify the uncertainty associated with FoldX prediction for individual mutations. Comparing the performance among models with varying degrees of complexity, we find that the model precision improves significantly when we utilize molecular dynamics simulation as part of the FoldX workflow. Based on the model that incorporates information from molecular dynamics, biochemical properties, as well as FoldX energy terms, we can generally expect upper bounds on the uncertainty of folding stability predictions of ± 2.9 kcal/mol and ± 3.5 kcal/mol for binding stability predictions. The uncertainty for individual mutations varies; our model estimates it using FoldX energy terms, biochemical properties of the mutated residue, as well as the variability among snapshots from molecular dynamics simulation.

**Conclusions:**

Using a linear regression framework, we construct models to predict the uncertainty associated with FoldX prediction of stability changes upon mutation. This technique is straightforward and can be extended to other computational methods as well.

**Supplementary Information:**

The online version contains supplementary material available at 10.1186/s12859-023-05537-0.

## Background

**Fig. 1:**
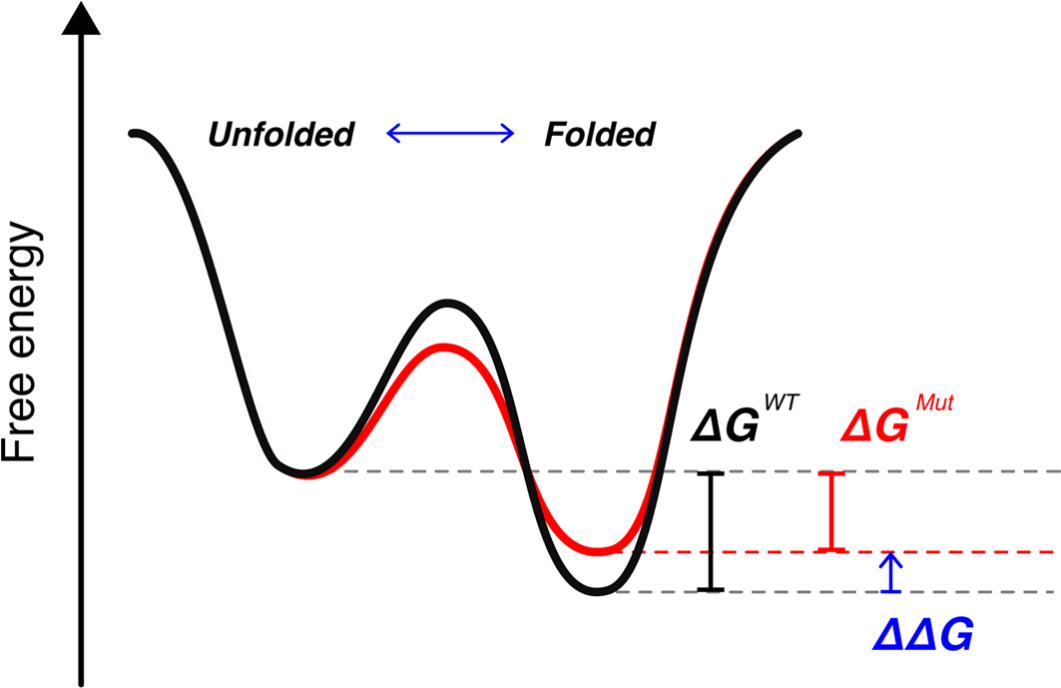
Visualization of protein folding energetics. $\mathrm{\Delta \Delta }G$ is the difference between ${\Delta G}^{WT}$ and ${\Delta G}^{Mutant}$, and informs the stability effect of a missense mutation. A mutation is destabilizing if it results in higher energy when folded than the wild-type, and stabilizing if the mutant achieves lower energy when folded than the wild-type. The energy curve colored in red illustrates a case of a destabilizing mutation

Computational methods that predict protein folding and binding stability are crucial in diverse areas of study ranging from molecular evolution to biomedicine. The changes in stability due to missense mutation can alter protein function, with implications for disease mechanisms and evolutionary trajectory. The ability to efficiently screen protein variants is also essential in protein engineering and drug discovery. Protein stability is quantified by a thermodynamic measure, Gibbs free energy ($\Delta G$), and the difference between $\Delta G$ of a wild-type and $\Delta G$ of a mutant ($\mathrm{\Delta \Delta }G$), indicates how strongly a mutation stabilizes or destabilizes folding or binding (Fig. 1).

While experimental measurement of $\Delta G$ is laborious and costly, numerous computational methods that predict protein stability are available and enable quick screening of a large number of protein variants. There are several methods of constructing force fields to calculate protein stability. Physical effective energy functions, also referred to as physical-based potentials, explicitly calculate energies based on the mechanics of physical forces between atoms. Statistical effective energy functions, or knowledge-based potentials, use statistical analysis of existing structure data [1–3]. A third category, empirical effective energy functions, is based on empirical data from experiments on single or multiple site mutations, and is the basis of the FoldX program that is the focus of this work [2, 4, 5]. Other methods, such as those utilizing machine-learning algorithms rather than explicit computation of energy contributions have also proliferated in recent years, adding to the wide array of tools available for in silico experiments in various fields [6–11].

FoldX is a popular tool due to the ease of use and computational speed. It calculates $\Delta G$ as a linear combination of contributing energy terms (see Table 2 for the list of energy terms) which is fitted to experimental data [2, 5]. After computing $\Delta G$ between unfolded and folded (or unbound and bound) states, $\mathrm{\Delta \Delta }G$ is then calculated as the difference between $\Delta G$ of the wild-type and $\Delta G$ of the mutant protein, i.e., $\mathrm{\Delta \Delta }G={\Delta G}^{Mutant}-{\Delta G}^{WT}$. In this definition, a negative $\mathrm{\Delta \Delta }G$ indicates stabilizing mutation while a positive $\mathrm{\Delta \Delta }G$ indicates destabilizing mutation.

FoldX has been an integral tool in our group’s body of work that combines molecular modeling and mutagenesis experiments [12–14]. However, a measure of uncertainty significantly improves the usefulness of the computational prediction. With the exception of MAESTRO, multi-agent machine-learning-based algorithm that returns a consensus predicted value along with confidence estimation [10], the only way to estimate the accuracy of most programs is through the published performance metrics available in literature. However, benchmarking results from different sources vary widely and are not likely to offer practical insight for a particular experiment. For example, the developers of FoldX reported the correlation between predicted $\mathrm{\Delta \Delta }G$ and experimentally-determined $\mathrm{\Delta \Delta }G$ to be 0.81 [5], but other studies have reported different levels of correlations, from as low as 0.19 to as high as 0.73 [15, 16]. In addition to this wide variation, interpreting these values is complicated by the fact that correlation depends not only on the error in FoldX but also on the range and distribution of $\mathrm{\Delta \Delta }G$ values among the mutations studied.

We also note that the error of an experimental measurement of $\mathrm{\Delta \Delta }G$ is another source of uncertainty. It is generally assumed that the experimental error is small, given identical experimental conditions, but can range from 0.1 to 0.5 kcal/mol [17]. While the issue of experimental error is not the focus of this study, it should be clear that this is a source of potential uncertainty. Here, we make the assumption that the experimental $\mathrm{\Delta \Delta }G$ values in databases represent the “truth.”

Our goal in this study is to develop a framework for quantifying the uncertainty associated with $\mathrm{\Delta \Delta }G$ prediction by FoldX. To that end, we constructed a set of linear regression models using datasets containing 1187 mutations (672 for folding stability and 515 for binding stability), generated prediction intervals around the point estimate of each individual mutation, and assessed model performance based on interval width and coverage. We built models across a spectrum of complexity: obtaining $\mathrm{\Delta \Delta }G$ by running FoldX on a single experimental protein structure vs on snapshot structures from a molecular dynamics (MD) simulation, and predictor variables ranging from FoldX energy terms to biochemical properties. We find that incorporating MD simulation greatly improves model performance, and that the uncertainty of FoldX is typically on the order of ± 3 kcal/mol for folding stability, and even larger for binding stability. The result further shows that we can infer the magnitude of the error based on major energy contribution terms in FoldX and biochemical properties intrinsic to the mutated residue. Our findings provide a more realistic interpretation of FoldX predictions and provide a framework that can be extended to other similar programs.

## Methods

### Datasets

Ten protein systems with experimentally-determined $\mathrm{\Delta \Delta }G$ of folding were selected from the ProTherm database [18] and ten protein complex systems with experimentally-determined $\mathrm{\Delta \Delta }G$ of binding from the Skempi database [19], as we have previously described [20]. The criteria for system selection included having both stabilizing and destabilizing mutations and having more than 20 mutations that were not all to alanine. Table 1 lists the selected systems along with their PDB IDs, the number of residues of the protein, the number of mutated residues, and number of mutations.

**Table 1:** PDB IDs and descriptions of the selected protein systems

Stability type	PDB ID	Total number of residues^a^	Number of mutated residues	Number of mutations
Folding	1BNI	110	67	163
	1BVC	153	37	56
	1HFZ	124	12	23
	1LZ1	130	53	116
	1PIN	163	32	56
	1RN1	104	23	48
	1RTB	124	22	50
	1VQB	87	34	92
	1WQ5	268	11	41
	2ABD	86	20	27
	Total	**–**	**–**	672
Binding	1A4Y	460/123	29	32
	1BRS	110/89	17	30
	1CBW	58	15	31
	1IAR	129	20	36
	1JTG	263/165	35	37
	1LFD	87	17	19
	1PPF	56	10	190
	2JEL	85	32	43
	2WPT	86/134	22	26
	3HFM	214/215/129	21	71
	Total	**–**	**–**	515

The dataset is comprised of 1187 mutations from 10 protein systems and 10 protein complexes for which experimental $\mathrm{\Delta \Delta }G$ values are known

^a^Sequence length as provided by RCSB Protein Data Bank. In case of protein complexes in binding stability dataset, a number is given for each mutagenized chain

### Obtaining predicted ${\varvec{\Delta}}{\varvec{\Delta}}\mathbf{G}$

The details of preparing structures, MD simulation, and FoldX analyses are described in Miller et al. [20]. To summarize, structure files downloaded from the PDB website using their PDB IDs (Table 1) were prepared for analysis by editing out unnecessary chains, fixing the missing residues, and standardizing the nomenclature. The edited structure files were then used to perform 100 ns-long MD simulations with the GROMACS 5.0.3 MD package to sample the configurational variation of proteins observed under physiological conditions. 100 snapshots, each 1 ns apart, were captured from each MD simulation. Each snapshot was then analyzed using FoldX 4.0 to calculate $\mathrm{\Delta \Delta }G$ of folding and binding upon mutations with available experimentally measured $\mathrm{\Delta \Delta }G$ values. $\mathrm{\Delta \Delta }G$ values per mutation from each of the 100 snapshots were then averaged to obtain the final $\mathrm{\Delta \Delta }G$. For comparison, we also built a set of analogous but separate datasets with $\mathrm{\Delta \Delta }G$ values calculated from using a single experimental structure file and analyzing with FoldX. Typically, a FoldX $\Delta G$ calculation output file contains the $\Delta G$ values associated with each energy term (Table 2). We used these output files to generate additional datasets with $\mathrm{\Delta \Delta }G$ values of individual energy terms resulting from FoldX calculations with MD snapshots and an experimental structure alone. Subsequent model selection and validation procedures described below were performed separately on these datasets.

**Table 2:** Predictor variables for the full model

FoldX energy terms (Refer to the online FoldX documentation for descriptions of these terms)		
Backbone van der Waals clash Cis bond Disulfide bond Electrostatic Electrostatic Kon	Entropy, main chain Entropy, side chain Helix dipole Hydrogen bond, backbone Hydrogen bond, sidechain Ionization	Solvation, hydrophobic Solvation, polar Torsional clash Total energy Van der Waals Van der Waals clashes
Biochemical properties		
Mutation involving proline: 0 or 1 Volume change: absolute difference between van der Waals volumes^a^ of the wild-type and the substituted amino acids in Å^3^ Hydrophobicity change: absolute difference between the hydrophobicity indices^b^ (at pH 7) of the wild-type and the substituted amino acids Charge change: absolute difference between the side chain changes of the wild-type and the substituted amino acids Secondary structure^c^: DSSP classification (B, E, G, H, I, S, T, or none) Relative solvent accessibility^d^: ranges from 0 (for completely buried) to 1 (completely exposed)		

Potential predictors in model search are all $\mathrm{\Delta \Delta }G$ terms (constituent energies and the total energy) output by FoldX and biochemical properties. The energy terms have standard deviation values that arise from averaging of MD snapshots and are used as predictor variables as well

^a^Zamyatnin [21]

^b^Monera et al. [22]

^c^Kabsch and Sander [23]

^d^Tien et al. [24]

### Defining variables

**Fig. 2:**
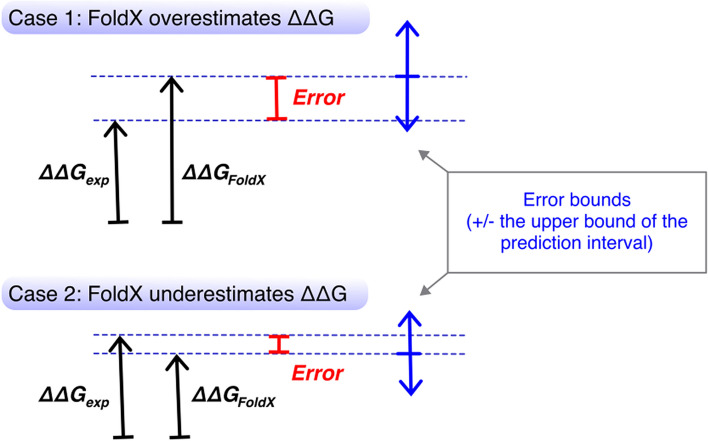
Conceptualization of *Error* and the error bound. We define *Error* as the absolute difference between ${\mathrm{\Delta \Delta }G}_{FoldX}$ and ${\mathrm{\Delta \Delta }G}_{exp}$ (Eq. 1). We construct a linear regression model that predicts the *Error*, and the model’s 95% prediction interval (the upper bound of the interval, specifically, since the prediction is an unsigned magnitude) captures ${\mathrm{\Delta \Delta }G}_{exp}$ (the “ground truth”) approximately 95% of the time

Our goal was to predict the uncertainty of the FoldX-calculated $\mathrm{\Delta \Delta }G$ (${\mathrm{\Delta \Delta }G}_{FoldX}$) assuming that the experimentally-determined $\mathrm{\Delta \Delta }G$ (${\mathrm{\Delta \Delta }G}_{exp}$) represents the truth. We defined this quantity, *Error* (Fig. 2), as the absolute difference between ${\mathrm{\Delta \Delta }G}_{FoldX}$ and ${\mathrm{\Delta \Delta }G}_{exp}$, and assigned it as the response variable in the model search.

1 $$Error=\left|{\mathrm{\Delta \Delta }G}_{FoldX}-{\mathrm{\Delta \Delta }G}_{exp}\right|$$

$\mathrm{\Delta \Delta }G$ of individual energy terms, e.g., van der Waals energy, solvation energy, entropy terms, etc. (Table 2), that contribute to the calculation of total ${\mathrm{\Delta \Delta }G}_{FoldX}$ were considered as potential predictor variables in the model search. This allows us to learn if an increase in any particular energy types correlates with increased error. When fitting models on the dataset generated from the MD snapshots where ${\mathrm{\Delta \Delta }G}_{FoldX}$ is an average from 100 snapshots, the standard deviation (*SD*) of ${\mathrm{\Delta \Delta }G}_{FoldX}$ was also included in the pool of predictors. A large *SD* indicates that a large amount of conformation variation is observed across the MD simulation, and may have an effect on the *Error*. We also considered biochemical properties of mutated residues such as secondary structures and solvent accessibility as potential predictors (Table 2).

### Model selection and evaluation

All model search process, validation, and subsequent analyses were performed using R 4.1. Two methods were used in the model selection: the stepwise selection method and the best subset selection method. In stepwise selection, predictor variables are alternatively added and removed one at a time until the best model is reached based on a selection criterion (i.e., all further steps produce models with poorer fit). On the other hand, best subset selection compares models with every possible combination of predictor variables. step function in stats package and regsubsets function in leaps package were used for these methods respectively. Both functions take a dataframe consisting of a response variable and all potential predictor variables as an input, perform the described search algorithm, and outputs the best model based on a given criterion. Bayesian information criterion (BIC) was our selection criterion of choice and is given by: $BIC=-2loglike+(\mathrm{log}\left(n\right))\bullet d$ where $loglike$ is the maximum log likelihood of the fitted model, $n$ is the number of observations, and $d$ is the number of predictor variables. BIC applies a heavy penalty for an increased number of parameters and thereby avoids overfitting.

We searched for five different types of models of varying levels of complexity, using the two selection methods (Table 3). For Model 1, the response variable, *Error*, is calculated based on a single experimental structure ${\mathrm{\Delta \Delta }G}_{FoldX}$, and the potential predictor variables made available to the model search were restricted to the individual energy terms from FoldX output (Table 2, “FoldX energy terms”). Model 2 is similar to Model 1 but the pool of predictor variables also included biochemical properties (Table 2, “Biochemical properties”). For Model 3, the response variable, *Error*, is calculated based on average ${\mathrm{\Delta \Delta }G}_{FoldX}$ from 100 MD snapshots. The potential predictor variables during model search were the individual energy terms from FoldX output (MD snapshot averages). Model 4 is similar to Model 3 but the potential predictors also included biochemical properties, and Model 5 also included *SD* values associated with each averaged energy term.

**Table 3:** Five types of resulting models

Model	Source of input structure to calculate $\mathrm{\Delta \Delta }G$	Predictor variables used in model search
Model 1	Single experimental structure	FoldX energy terms
Model 2	Single experimental structure	FoldX energy terms and biochemical properties
Model 3	MD snapshots	FoldX energy terms
Model 4	MD snapshots	FoldX energy terms and biochemical properties
Model 5 (full model)	MD snapshots	FoldX energy terms and their associated *SD*, and biochemical properties

We constructed and tested models with various levels of complexity. Models 1 and 2 were based on datasets with $\mathrm{\Delta \Delta }G$ calculated from a single experimental structure while Models 3–5 were based on MD snapshot averages. We also varied the types of predictor variables.

We compared the model performance using a modified leave-one-out cross validation, a common method in which the *i*-th observation in a dataset is excluded from model training. The model fitted with $n-1$ observations is then tested on the excluded datapoint. This process is repeated to obtain $n$ testing results and the aggregate is an indication of the overall model performance. This can be in the form of average mean squared error in cases involving continuous response variables, or an error rate in classification problems. While our model predicts *Error* that is on a continuous scale, we used the upper bound of the 95% prediction interval (Fig. 2) to calculate coverage as a performance metric. Also, because we were concerned about nonindependence among data from the same system and suspected qualitative differences among the systems, we modified the method by excluding all datapoints from one system as a testing set—i.e., we leave one system out. The candidate model is fit using the rest of the datapoints from the remaining nine systems, and tested on the excluded datapoints. This process is repeated for each system and the coverage of a model is calculated as follows:

2 $$Coverage=\frac{1}{N}\sum_{i=1}^{10}\sum_{j=1}^{{n}_{i}}I\left({Error}_{j}<{B}_{j}\right)$$

where $N$ is the total number of observations in the dataset, ${n}_{i}$ is the number of observations in $i$-th system, and ${B}_{j}$ is the upper bound of the prediction interval of the *Error* at the $j$-th observation in the $i$-th system. The magnitude of the upper bound is an indication of the model precision, and we used the median width (to avoid over-influence of large values) as a part of the performance metric. All together, we produced and evaluated a total of ten models: one set of five models (Table 3) using the folding stability dataset, and another set using the binding stability dataset.

## Results

### Leave-one-system-out cross validation result

With datasets comprising of single amino acid mutations with known experimental $\mathrm{\Delta \Delta }G$ (${\mathrm{\Delta \Delta }G}_{exp}$) and FoldX-predicted $\mathrm{\Delta \Delta }G$ (${\mathrm{\Delta \Delta }G}_{FoldX}$), we used stepwise selection and best subset selection methods of model search to produce five pairs—one from each selection method—of different models (Table 3) for each of the folding dataset and binding dataset. We then determined the better model from each pair by *leave-one-system-out* cross validation where the model is fit with data from nine protein systems and tested on the remaining datapoints iteratively. The coverage rate from this process (Eq. 2) is the proportion of the prediction intervals that capture the *Error* when tested on the data the model was not trained on. Based on this measure of model accuracy and the median interval widths, a measure of model precision, we found that models from the best subset selection method performed better than those from stepwise selection method. We tested backward elimination as well but further removal of predictor variables did not improve performance when tested with cross validation.

After we determined the final five sets of models (Table 3) for each of the folding and binding datasets, we assessed the performance among them. Comparing models built with single-structure $\mathrm{\Delta \Delta }G$ (Models 1 and 2) and those with MD snapshots (Models 3–5), coverage was similar in all cases at approximately 95%. However, the median width of the prediction interval decreased considerably in Models 3–5, especially in the folding dataset (Table 4). This implies that the additional information supplied by MD simulation brings a marked improvement in model precision. This is also evidenced by the large decrease in BIC between Models 1–2 and Models 3–5 (Table 4). Subsequent results and discussions will focus on the full model (Model 5), but we emphasize that qualitative results among shared variables are similar in all models.

**Table 4:** Comparison of model performances

	Model 1	Model 2	Model 3	Model 4	Model 5 (full)
Folding dataset:					
Coverage (%)	94.5	94.5	94.5	94.3	94.6
Median width (kcal/mol)	3.78	3.75	2.88	2.84	2.89
Adjusted *R*^*2*^	0.17	0.22	0.42	0.45	0.44
BIC	2279	2284	1928	1936	1930
Binding dataset:					
Coverage (%)	94.6	95.7	95.1	94.6	94.8
Median width (kcal/mol)	3.81	3.74	3.52	3.51	3.47
Adjusted *R*^*2*^	0.62	0.66	0.52	0.56	0.57
BIC	1814	1813	1710	1709	1700

*Leave-one-system-out* cross validation results show all models achieve approximately 95% coverage. Median widths decreased in Models 3–5 which used MD snapshots data. While *R*^*2*^ values were referenced during evaluation, more emphasis was given to the cross validation results (coverage and median width.)

**Fig. 3:**
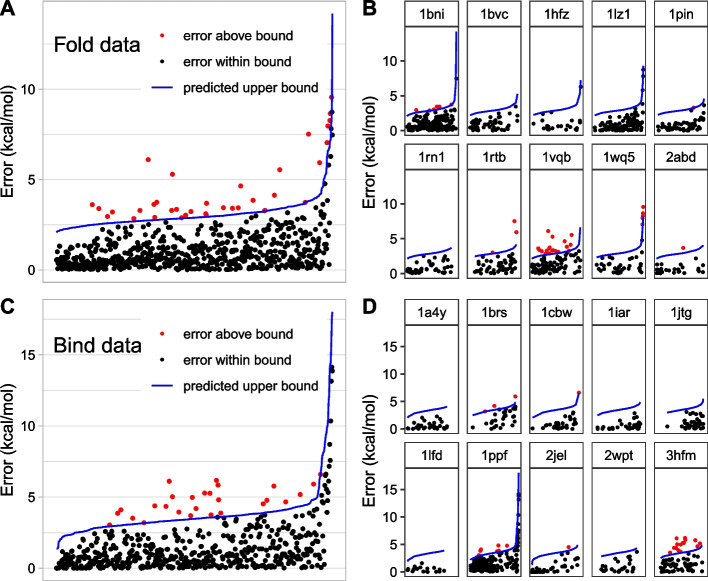
Coverage of the full model. The *Error* of each mutation is sorted by the width of the prediction interval (blue line) and plotted. Datapoints are colored red if they were outside of the bound. Approximately 95% of the datapoints are captured within the bound when considered all together. **A** All 672 mutations in the folding stability dataset. **B** System-by-system breakdown of A. **C** All 515 mutations in the binding stability dataset. **D** System-by-system breakdown of **C**

While the models achieve 95% coverage overall, a closer analysis of the cross validation results reveals that the coverage varies widely among protein systems as shown in Fig. 3 (panels B and D), and Table 5. For the folding energy dataset, there were a total of 36 outliers—i.e., those not captured by the prediction interval—out of 672 datapoints and 19 of them were mutations in 1VQB, 7 in 1BNI, and 5 in 1WQ5 (*p*-value = 0.00050, chi-squared goodness-of-fit test). Out of 27 outliers for the binding energy dataset, 15 were from 3HFM, and 4 from 1PPF (*p-*value = 0.00050, chi-squared goodness-of-fit test).

**Table 5:** System-by-system breakdown of the model performance

Energy type	PDB ID	Mutations	Median Error	Coverage (%)	Median width
Fold	1BNI	163	0.63	96	2.90
	1BVC	56	0.70	100	2.73
	1HFZ	23	0.63	100	2.93
	1LZ1	116	0.75	100	3.08
	1PIN	56	0.53	98	2.94
	1RN1	48	0.55	100	2.72
	1RTB	50	1.00	94	3.03
	1VQB	92	1.28	79	2.77
	1WQ5	41	1.13	88	2.93
	2ABD	27	0.52	96	2.77
	Total	672	0.73	95	2.89
Bind	1A4Y	32	0.35	100	3.46
	1BRS	30	2.27	90	3.78
	1CBW	31	0.84	97	3.65
	1IAR	36	0.30	100	3.89
	1JTG	37	0.94	100	3.99
	1LFD	19	0.39	100	3.13
	1PPF	190	1.17	96	3.29
	2JEL	43	0.17	98	3.05
	2WPT	26	0.54	100	3.53
	3HFM	71	1.40	79	3.46
	Total	515	0.90	95	3.47

Inspection of the model performance (full model) separately for each protein system reveals a wide variation

### Significant predictors

For the folding energy dataset, the predictors of the final models are van der Waals energy, van der Waals clash, side chain entropy, *SD* of total energy, involvement of proline, and secondary structure. For binding, the predictors are van der Waals clash, *SD* of backbone clash, *SD* of side chain entropy, *SD* of total energy, secondary structure, and relative solvent accessibility (RSA). Table 6 lists the predictor variables and their statistical details. Details for the entire ten models are available in the Additional File 1. Table 6 also shows the effect of each predictor on the prediction interval width (see details in table legend).

**Table 6:** Significant predictors in full models and their statistical details

Energy type	Predictor	Coefficient estimate	*p *value	Effect on interval width
Fold	Van der Waals	0.631	5.67e−14	1.241
	Van der Waals clash	0.431	< 2e−16	0.705
	Entropy, side chain	0.531	3.16e−7	0.775
	*SD* of total energy	0.569	8.55e−6	0.519
	Mutation involving proline	0.724	2.08e−4	0.761
	Secondary structure—B	Reference	N/A	N/A
	Secondary structure—E	0.239	0.375	N/A
	Secondary structure—G	0.069	0.847	N/A
	Secondary structure—H	− 0.181	0.499	N/A
	Secondary structure—None	0.026	0.924	N/A
	Secondary structure—S	0.144	0.633	N/A
	Secondary structure—T	− 0.148	0.604	N/A
Bind	Van der Waals clash	0.418	3.28e−5	2.531
	*SD* of backbone van der Waals clash	6.69	3.63e−4	0.864
	*SD* of van der Waals clash	− 1.06	5.69e−4	− 2.518
	*SD* of entropy, side chain	− 1.21	9.08e−4	− 0.748
	*SD* of total energy	1.32	4.85e−6	3.791
	Secondary structure—B	Reference	N/A	N/A
	Secondary structure—E	0.240	0.655	N/A
	Secondary structure—G	− 0.617	0.434	N/A
	Secondary structure—H	0.116	0.830	N/A
	Secondary structure—None	− 0.236	0.659	N/A
	Secondary structure—S	0.336	0.547	N/A
	Secondary structure—T	0.250	0.650	N/A
	RSA	− 1.45	3.51e−7	− 0.977

The coefficients and *p*-values are from the model fitted to all datapoints. The effect of each predictor on interval width (right column) is a rescaled version of the coefficient. It is the difference in interval width between two hypothetical mutations—one representing the upper and one the lower 10% quantile of the predictor—while holding all other predictors constant. More precisely, for each predictor, we generated two contrasting mutations with predictor values equal to the mean of the predictor among the upper and lower 10% quantiles, set all other predictor values to their dataset-wide averages, used the predict() function in the model to predict the upper bound on their error, and took the difference. In the case of the proline predictor, the contrasting mutations were with and without proline (with everything else at their averages)

## Discussion

### A novel approach to estimate the error of predicted ${\varvec{\Delta}}{\varvec{\Delta}}\mathbf{G}$

This study seeks to quantify the uncertainty associated with computational prediction of $\mathrm{\Delta \Delta }G$ to aid researchers in informed usage of the prediction. Specifically, we focused on FoldX because it is one of the popular and readily-available software. We built multiple linear regression models with predictor variables selected from a pool of FoldX energy terms and biochemical properties to predict the errors associated with each ${\mathrm{\Delta \Delta }G}_{FoldX}$ value. We assessed models of varying complexity using an empirical test of *leave-one-system-out* cross validation and determined that the performance improves considerably in models trained on datasets based on ${\mathrm{\Delta \Delta }G}_{FoldX}$ from MD snapshots (Models 3–5). The coefficients (sign and rank) of the shared predictors among these models were qualitatively similar. Furthermore, the full model had the best BIC score for binding ($\Delta BIC$ = -9) and was similar to Model 3 for folding ($\Delta BIC$ = + 2). Given this, we focused on the full model that contains the most diverse sets of predictors in order to explore the effects of the FoldX energy terms, MD simulation, and biochemical properties of the mutated residues, on estimating the error.

One of our major findings is that the margin of error in ${\mathrm{\Delta \Delta }G}_{FoldX}$ is much larger than the general assumption in the field. According to the cross validation results of Models 3–5 which utilize MD simulation, intervals of approximately ± 2.9 kcal/mol for folding stability and ± 3.5 kcal/mol for binding stability around a given ${\mathrm{\Delta \Delta }G}_{FoldX}$ are necessary in order to capture ${\mathrm{\Delta \Delta }G}_{exp}$ within 95% prediction interval. The intervals are even wider for models based on single-structure calculation (i.e., no MD simulation). Given this wide margin, researchers should be cautious in how FoldX predictions are utilized.

While the median interval width provides a general idea of potential errors, the intervals can be smaller or larger based on the unique characteristics of the mutated residue and the nature of the amino acid substitution. The mutation-specific estimated error can then be used as a weighting factor in downstream usage of ${\mathrm{\Delta \Delta }G}_{FoldX}$. The conventional way of error estimation is limited to the blanket rule-of-thumb based on published software performance data, which has a wide range as pointed out previously. While the developers of FoldX reported a correlation of 0.81 with *SD* of 0.46 kcal/mol between ${\mathrm{\Delta \Delta }G}_{exp}$ and ${\mathrm{\Delta \Delta }G}_{FoldX}$ based on more than 1000 mutations [5], other studies have reported lower correlations: 0.54 based on a curated 605 mutations [25] and 0.50 based on another curated set of 1200 mutations [3]. Much lower correlations—as low as 0.19—come from studies that tested FoldX on a limited set of mutations from a single protein [15, 26]. In addition to the wide variation of reported accuracy, another limitation is that a summary measure such as the correlation coefficient does not fully inform the extreme ends of the variability in error.

Some studies suggest FoldX performs better as a qualitative predictor. In the aforementioned study by Potapov et al., the accuracy (percentage of correctly predicted stabilizing or destabilizing mutations out of all mutations) of FoldX was 69.5% based on 1200 mutations with the classification determined by whether $\mathrm{\Delta \Delta }G$ was greater or less than 0 kcal/mol. Accuracy increased to 74.2% when only the mutations with $\left|\mathrm{\Delta \Delta }G\right|>$ 2 kcal/mol were considered [3]. However, FoldX has a tendency to predict destabilizing mutations with higher accuracy than stabilizing mutations. This biased prediction accuracy is a well-known drawback of many computational programs, arising from the fact that a random mutation tends to be destabilizing and thus most training datasets are comprised of mostly destabilizing mutations [27–29].

**Fig. 4:**
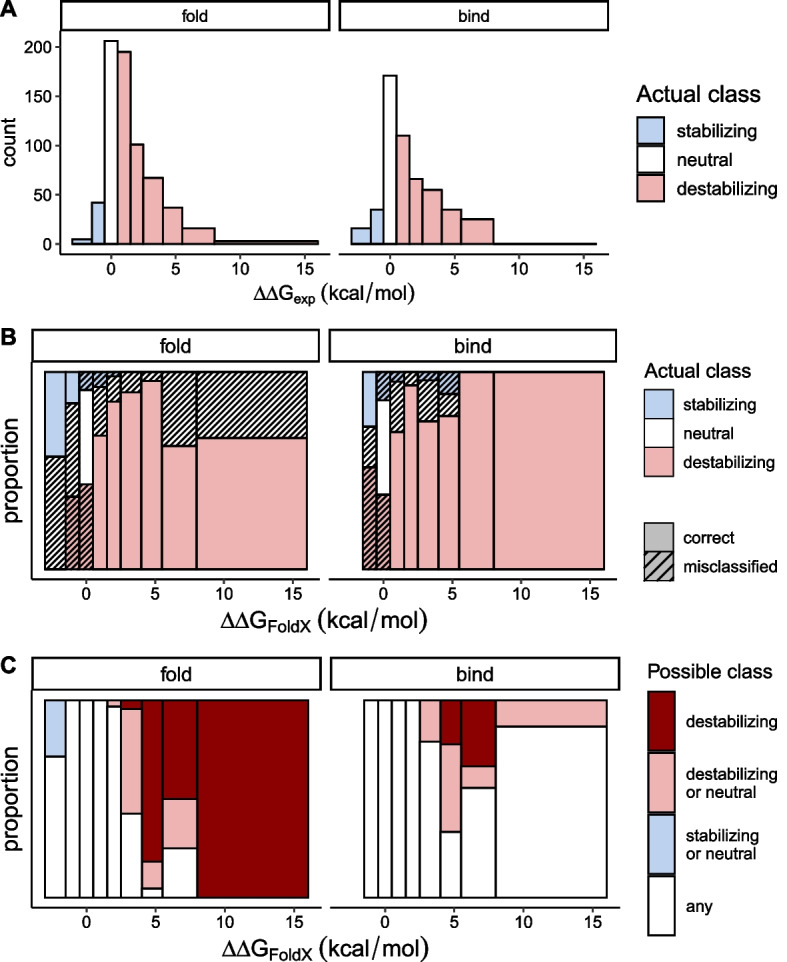
Analysis of classification errors. **A** Histograms of 672 mutations in folding energy dataset and 515 mutations in binding energy dataset show the unequal distributions of stabilizing (${\mathrm{\Delta \Delta }G}_{exp}<$ − 0.5 kcal/mol), neutral (between − 0.5 and 0.5 kcal/mol), and destabilizing (> 0.5 kcal/mol). **B** The relative frequencies of the stabilizing, neutral, and destabilizing mutations are plotted along intervals of ${\mathrm{\Delta \Delta }G}_{FoldX}$. Shaded areas indicate incorrect prediction by FoldX. FoldX tends to predict destabilizing mutations correctly than stabilizing mutations. **C** Same sets of mutations are classified according to the interval ${\mathrm{\Delta \Delta }G}_{FoldX}$ ± error bounds, and their relative frequencies are expressed along ${\mathrm{\Delta \Delta }G}_{FoldX}$

The overall accuracy of qualitative prediction is mostly driven by the fact that FoldX tends to correctly predict destabilizing mutations that often make up a larger part of the training dataset as in our case (Fig. 4A). In panel B, we can see that more than 75% of the mutations predicted to be destabilizing are correctly identified (pink, non-shaded areas). In contrast, more than half of the mutations which FoldX predicted to be stabilizing ($\mathrm{\Delta \Delta }G<$ -0.5 kcal/mol, the two leftmost bins) are actually neutral or destabilizing (shaded white and pink areas). In Fig. 4C, where the colors indicate possible classification based on our error bounds instead of ${\mathrm{\Delta \Delta }G}_{exp}$, we can see that most of the mutations near zero (${\mathrm{\Delta \Delta }G}_{FoldX}$ ±2 kcal/mol) cannot be classified with confidence. With folding dataset, we can be more confident with mutations whose ${\mathrm{\Delta \Delta }G}_{FoldX}>$ 2.5 kcal/mol to be truly destabilizing. The unexpected trend in binding dataset where the uncertainty increases again, with ${\mathrm{\Delta \Delta }G}_{FoldX}>$ 8 kcal/mol in particular, is due to all of the 15 mutations in that bin belonging to a single protein system, 1PPF, having an unusually large ${\mathrm{\Delta \Delta }G}_{FoldX}$ and error bounds.

**Fig. 5:**
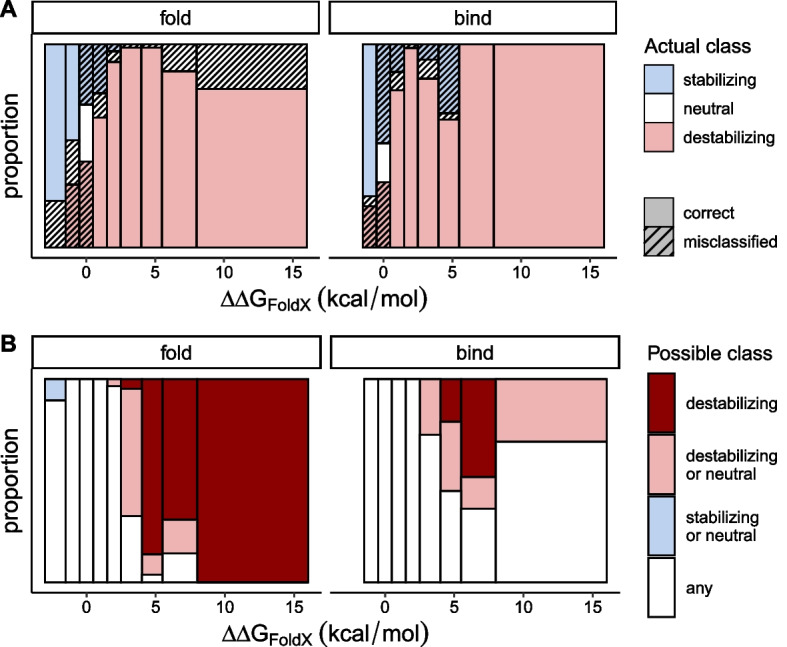
Analysis of classification errors with resampling. **A** After resampling 1000 observations from each interval of ${\mathrm{\Delta \Delta }G}_{exp}$, the relative frequencies of the stabilizing, neutral, and destabilizing mutations are plotted along intervals of ${\mathrm{\Delta \Delta }G}_{FoldX}$. Shaded areas indicate incorrect prediction by FoldX. **B** Same sets of resampled mutations are classified according to the interval, ${\mathrm{\Delta \Delta }G}_{FoldX}$ ± error bounds, and their relative frequencies are expressed along ${\mathrm{\Delta \Delta }G}_{FoldX}$

We were next interested in identifying the extent to which the type of mutation (stabilizing vs destabilizing) affects the misclassification problem. The misclassification rates seen in Fig. 4B are influenced by the underlying distribution of $\mathrm{\Delta \Delta }G$ for the mutations chosen in a particular study as well as the variance and bias of the prediction method, such as FoldX. Specifically, the low proportion of correctly-identified stabilizing mutations in Fig. 4B is a result of the inherent bias of FoldX *and* the low frequency of stabilizing mutations in our data. In order to understand the effect of the underlying distribution, we created a dataset by resampling (with replacement) 1000 observations from each bin along ${\mathrm{\Delta \Delta }G}_{exp}$, essentially simulating a scenario of picking mutations from a uniform distribution of ${\mathrm{\Delta \Delta }G}_{exp}$. When we visualize the proportion of correctly-predicted classifications, we see similar accuracy among stabilizing and destabilizing mutations (Fig. 5A). Essentially, a uniform sampling of ${\mathrm{\Delta \Delta }G}_{exp}$ yields high classification accuracy among mutations observed to be strongly stabilizing and destabilizing. However, misclassification remains a problem for mutations with ${\mathrm{\Delta \Delta }G}_{exp}$ values near zero. As Fig. 5B shows, only those predicted to be strongly destabilizing can be classified unambiguously, which was also the case before resampling (Fig. 4C). Altogether, we find that the uncertainty of FoldX prediction is large enough that classifying mutations based on ${\mathrm{\Delta \Delta }G}_{FoldX}$ is not reliable.

### Predictor variables and their role

In addition to providing a measure of uncertainty of individual ${\mathrm{\Delta \Delta }G}_{FoldX}$ values, our model also gives an insight into the factors that influence that uncertainty. In their publication describing the development of the energy function used in the FoldX program, Guerois et al. conducted a detailed analysis of outlier mutations both in training database and test database. Their explanations for the large discrepancy between predicted and experimental $\mathrm{\Delta \Delta }G$ include mutations that greatly affect the unfolded state, possible structural relaxation from removal of large side chains (most of these are mutations to alanine or glycine), and mutations from proline. Some of these outliers originate from a specific protein (1FMK in the training database and 1STN in the test database) and their analysis surmised that these discrepancies were not generalized [2]. While we also observe system-specific differences in median *Error* and the model’s predictive accuracy (Fig. 3 and Table 5), our model shows the *Error* is influenced by factors that are generalizable across protein systems.

For the folding energy dataset, van der Waals and van der Waals clash terms appear consistently with significant effects in all five models. Holding other variables equal, having high values for these predictors cause a significant increase in uncertainty, as can be seen in the Coefficient estimates and the Effects on interval width in Table 6. Mechanistically, van der Waals energy arises from the electrostatic forces between atoms, relative to the distance between them. In the FoldX algorithm, van der Waals is determined by the transfer energy of the molecule from vapor to water. Increased van der Waals implies a large change in inter-atom forces as a residue is substituted. It may be that the larger this change, the more difficult and error-prone it becomes to predict the stability. Similarly, amino acid substitutions that result in a large change in van der Waals clash appear to increase *Error* as well. Clashes occur when there is an overlap of atomic radii such as when a small amino acid is mutated to a large amino acid. While FoldX can compute local rearrangement around the mutated residue, it cannot account for the more global rearrangement in the protein that may occur, leading to an inaccurate prediction. This may also be the reason that using MD snapshots tends to improve FoldX prediction since the protein can change conformation near the mutation site. Van der Waals clash in FoldX is a corrective term to compensate for the overestimation of solvation and van der Waals energies arising from a steric clash [2]. Large clash values thus imply that energies are calculated from incorrect atomic positions and the compensation by the clash term may not be adequate for some mutations.

The effect of another significant predictor, side chain entropy, may follow a similar reasoning. The side chain entropy represents the entropic cost of fixing the side chain upon folding (or binding) —i.e., unfavorable $\Delta G$ contribution due to the decrease in the conformational space available to the side chain arrangement. A large difference in $\Delta S$ (entropy) between the wild-type and the mutated residue implies a severe steric restriction such as when a bulky amino acid replaces a small one. As with van der Waals energy and van der Waals clash terms, the accuracy of FoldX seems to decline when it computes a large change in entropy.

*SD* of total energy indicates the variation among $\mathrm{\Delta \Delta }G$ of total energy calculated for each of the 100 MD snapshots, and is a significant predictor for the folding energy model. For binding energy, several *SD* terms, including van der Waals clash, backbone van der Waals clash and side chain entropy, appear significant and their contribution to the model performance is evidenced by the lowest BIC values (Table 4). Structures that exhibit large fluctuations of coordinates during MD simulation produces a large *SD* of the ${\mathrm{\Delta \Delta }G}_{FoldX}$ values calculated from those snapshots. It may mean that $\mathrm{\Delta \Delta }G$—whether experimental or computational—is difficult to measure accurately for mutated residues occurring in the regions with high conformational variability. Other *SD* terms in the binding model have negative effects on *Error* (larger the *SD*, smaller the *Error*), making it difficult to deduce the mechanism behind the observation. We can only surmise that the degree of conformational variation impacts the accuracy of FoldX calculation.

As for biochemical properties, a notable predictor is proline—either a proline residue in wild-type or a mutation to proline. With all other variables being equal, the involvement of proline increases the uncertainty (i.e. interval width) by approximately 0.761 kcal/mol (Table 6). The cyclic structure of proline constricts bond angles and may disrupt the secondary structure of a peptide chain or introduce steric clashes, resulting in inaccurate ${\mathrm{\Delta \Delta }G}_{FoldX}$. Mutations involving glycine and alanine are also known to affect stability prediction and were tested during model search. However, in the presence of a larger pool of potential predictors (energy terms, etc.), only proline was significant enough to remain in the final models. We verified that this result was not due to sample sizes as the number of mutations involving proline (n = 27) was smaller than that of alanine (n = 251) or glycine (n = 105). The significance of proline mutations in spite of the small number of datapoints indicates that the unique biochemistry of proline makes accurate prediction of $\mathrm{\Delta \Delta }G$ challenging.

The main driver of protein folding is hydrophobic packing where hydrophobic residues aggregate in the core and minimize their exposure to the polar solvent. Since a mutation of a core residue is more likely to disrupt the stability of the folded protein than a surface residue, we suspected that RSA, which measures how exposed or buried a residue is, might have a role in estimating the *Error*. Interestingly, RSA appeared only with the binding energy dataset, with a negative effect. A mutation occurring at a residue that has high RSA—i.e., more exposed and away from the binding interface—will not change $\mathrm{\Delta \Delta }G$ significantly and therefore will have less error associated with the prediction. It appears that this effect of RSA on the *Error* is not as pronounced with $\mathrm{\Delta \Delta }G$ of folding stability in the presence of many other potential predictors as with $\mathrm{\Delta \Delta }G$ of binding stability.

The role of secondary structure as a predictor is less straightforward. We treated the secondary structure as a categorical variable with seven levels following the DSSP classification scheme [23], excluding I which is a rare $\pi$-helix and not present in our datasets. We suspected that highly organized structures such as helices or $\beta$ sheets (H, G, E) might show a significant contribution to the *Error* compared to less rigid structures such as loops (S, T), but did not see this pattern in our result. In spite of the unclear directionality of their effects and high *p*-values (Table 6), eliminating this predictor from the model resulted in inferior performance in cross validation.

### Leave-one-system-out cross validation to account for the variation among protein systems and complexes

The applicability of this study is twofold: (1) we learn the factors that influence the *Error* as discussed in the previous section, and (2) we can apply our trained models to a new protein system or complex in which we do not have experimental data on stability. To achieve the second point, it was critical that we avoid overfitting and prioritize generalizability while keeping the loss of precision as minimal as possible. We used BIC that applies a heavier penalty on the number of parameters than AIC during the model search, and relied on the *leave-one-system-out* cross validation to determine the best-performing model rather than on BIC alone.

The result of this cross validation method simulates a real-world scenario of applying a model trained on a dataset with known ${\mathrm{\Delta \Delta }G}_{exp}$ to a new set of mutations—most likely in a protein that was not in the training data—where ${\mathrm{\Delta \Delta }G}_{exp}$ is unknown. The system-by-system breakdown in Fig. 3 and Table 5 provides a glimpse of the best-case and worst-case scenarios. For example, we can expect coverage greater than 95% for most systems (7 out of 10 for folding stability and 8 out of 10 for binding stability). It may be as low as less than 80% in a minority of cases (as in 1VQB for folding energy and 3HFM in binding energy). The model precision (prediction interval width) also varies among systems but not as widely as the coverage.

The uneven distribution of the model accuracy (in terms of coverage) suggests there are system-specific factors that we are not able to incorporate in our models. Notwithstanding, the utility of the model is in a comparative analysis of mutations: given a group of mutations with FoldX predictions, the error bounds can alert the user to particular mutations whose true ${\mathrm{\Delta \Delta }G}_{exp}$ are likely to deviate greatly from ${\mathrm{\Delta \Delta }G}_{FoldX}$ based on the mutation-specific characteristics.

### The role of molecular dynamics simulation

While MD is not a part of conventional FoldX workflow, it has been shown that averaging of $\mathrm{\Delta \Delta }G$ from MD snapshots results in an improved correlation between ${\mathrm{\Delta \Delta }G}_{FoldX}$ and ${\mathrm{\Delta \Delta }G}_{exp}$ [20, 30]. In order to test whether the improvement extends to the error estimation, we built models separately using datasets with $\mathrm{\Delta \Delta }G$ calculated from a single experimental structure (Models 1–2) and datasets with averaged $\mathrm{\Delta \Delta }G$ from MD snapshots (Models 3–5). While all models maintained the coverage rate of approximately 95%, the median widths of the prediction interval noticeably decreased in the models with MD snapshots data (Table 4). The rationale for MD simulation stems from the fact that a single structure of a protein does not accurately represent the true conformational space in a natural, aqueous environment, and hence inaccurate ${\mathrm{\Delta \Delta }G}_{FoldX}$. MD snapshots sample various conformations of the protein, and the average ${\mathrm{\Delta \Delta }G}_{FoldX}$ across these structures take this variation into account. Implicit in the *SD* values associated with each averaged ${\mathrm{\Delta \Delta }G}_{FoldX}$ is the degree of the conformational variability, and we found that they are significant predictors of the *Error* (Table 6). We also saw that MD simulation contributes to the model precision, as the MD snapshots-based models showed tighter prediction intervals than the single-structure-based models (Table 4). However, we are unable to delineate whether the extra information from MD simulation directly contributes to the improved precision or if it only decreases the *Error* itself and the tighter interval is a consequence of the smaller *Error* estimate.

Altogether, the models are able to predict potential errors with much better precision when MD simulation is utilized. We recognize that MD simulation is a technical resource that may not be available readily. Even without it, we have shown that models can be built from single-structure datasets with comparable coverage albeit with wider prediction intervals. An addition of biochemical properties, that can be easily calculated, improves the precision slightly by shortening the intervals.

## Conclusions

Computational methods of predicting mutational effects on protein stability are invaluable in high-throughput mutational studies. While there are abundant benchmark studies on the performance of various methods, few programs offer a measure of uncertainties associated with individual predictions. Focusing on the popular program, FoldX, we built multiple linear regression models to predict the magnitude of the discrepancy between the experimental and computational stability changes due to single amino acid mutations. We found that the model performs best when supplied with information from MD simulation of the protein and biochemical properties of the mutated residues. However, simpler models based on only FoldX information can still be useful. Our models also provided mechanistic insight into the factors that contribute to the error. Because our models predict errors based on the mutation-specific predictors, the unique error estimates can then be used as weighting factors in downstream analyses using FoldX results. This method can be extended beyond FoldX and has the potential to be a tool for researchers to help guide their computational predictions.

### Supplementary Information

**Additional file 1.** An R output file showing a performance comparison summary of all models and model details of each, listing the significant predictors.

## Data Availability

Datasets and the code to generate results in the paper are available at https://github.com/yesols/foldx_uncertainty, archive https://doi.org/10.5281/zenodo.7897628. FoldX program is publicly available from their website: https://foldxsuite.crg.eu/. DSSP program for obtaining secondary structure information is available at: https://swift.cmbi.umcn.nl/gv/dssp/. PDB files for all twenty protein systems were downloaded from RCSB Protein Data Bank website: https://www.rcsb.org/.

## Abbreviations

BIC: Bayesian information criterion
MD: Molecular dynamics
RSA: Relative solvent accessibility
SD: Standard deviation
