# Supplementary material for: Statistical modeling to quantify the uncertainty of FoldX-predicted protein folding and binding stability
Source: BMC Bioinformatics. 2023 Nov 12;24:426. doi: 10.1186/s12859-023-05537-0 (PMC10642056; doi:10.1186/s12859-023-05537-0)
Supplement: Supplementary file 1 — Additional file 1. An R output file showing a performance comparison summary of all models and model details of each, listing the significant predictors. [file 12859_2023_5537_MOESM1_ESM.html]

Supplementary Information: Model Summary


# Supplementary Information: Model Summary

#### Yesol Sapozhnikov

## Model performance comparison

Fold dataset

```
##   models    adj_r2  coverage med_upr_bd
## 1   mod1 0.1719845 0.9449405   3.778818
## 2   mod2 0.2183231 0.9449405   3.745220
## 3   mod3 0.4190675 0.9449405   2.878458
## 4   mod4 0.4450158 0.9434524   2.843904
## 5   mod5 0.4410874 0.9464286   2.889315
```

Bind dataset

```
##   models    adj_r2  coverage med_upr_bd
## 1   mod1 0.6247946 0.9456311   3.810007
## 2   mod2 0.6581134 0.9572816   3.736435
## 3   mod3 0.5197584 0.9514563   3.517148
## 4   mod4 0.5580397 0.9456311   3.509464
## 5   mod5 0.5663188 0.9475728   3.471100
```

## Model details: Fold

### Model 5 (MD+FX dataset with all predictors)

```
## 
## Call:
## lm(formula = f, data = datasets[[i]])
## 
## Residuals:
##     Min      1Q  Median      3Q     Max 
## -3.4991 -0.5770 -0.1423  0.4294  5.2028 
## 
## Coefficients:
##                 Estimate Std. Error t value Pr(>|t|)    
## (Intercept)      0.53656    0.26169   2.050 0.040725 *  
## energy_vdw       0.63061    0.08209   7.682 5.67e-14 ***
## energy_vdwclash  0.43121    0.04720   9.137  < 2e-16 ***
## entropy_sidec    0.53108    0.10279   5.167 3.16e-07 ***
## total_sd         0.56925    0.12689   4.486 8.55e-06 ***
## P                0.72364    0.19398   3.730 0.000208 ***
## strE             0.23919    0.26946   0.888 0.375050    
## strG             0.06877    0.35639   0.193 0.847036    
## strH            -0.18056    0.26674  -0.677 0.498693    
## strNONE          0.02617    0.27434   0.095 0.924038    
## strS             0.14408    0.30131   0.478 0.632686    
## strT            -0.14754    0.28400  -0.520 0.603575    
## ---
## Signif. codes:  0 '***' 0.001 '**' 0.01 '*' 0.05 '.' 0.1 ' ' 1
## 
## Residual standard error: 0.9636 on 660 degrees of freedom
## Multiple R-squared:  0.4502, Adjusted R-squared:  0.4411 
## F-statistic: 49.14 on 11 and 660 DF,  p-value: < 2.2e-16
```

### Model 4 (MD+FX dataset with energy terms and biochemical properties)

```
## 
## Call:
## lm(formula = f, data = dataset)
## 
## Residuals:
##     Min      1Q  Median      3Q     Max 
## -4.0967 -0.5747 -0.1561  0.4343  5.1831 
## 
## Coefficients:
##                 Estimate Std. Error t value Pr(>|t|)    
## (Intercept)      0.79435    0.25746   3.085 0.002119 ** 
## energy_solvh    -0.47413    0.12935  -3.666 0.000267 ***
## energy_vdw       1.00020    0.21614   4.628 4.46e-06 ***
## energy_vdwclash  0.33131    0.07397   4.479 8.86e-06 ***
## entropy_mainc   -0.49116    0.11289  -4.351 1.57e-05 ***
## entropy_sidec    0.58026    0.11387   5.096 4.55e-07 ***
## total            0.26883    0.06521   4.122 4.23e-05 ***
## P                0.77331    0.19492   3.967 8.07e-05 ***
## strE             0.26054    0.26855   0.970 0.332311    
## strG            -0.06144    0.35734  -0.172 0.863537    
## strH            -0.19846    0.26678  -0.744 0.457203    
## strNONE         -0.02394    0.27428  -0.087 0.930466    
## strS             0.11887    0.30054   0.396 0.692578    
## strT            -0.17152    0.28450  -0.603 0.546787    
## ---
## Signif. codes:  0 '***' 0.001 '**' 0.01 '*' 0.05 '.' 0.1 ' ' 1
## 
## Residual standard error: 0.9602 on 658 degrees of freedom
## Multiple R-squared:  0.4558, Adjusted R-squared:  0.445 
## F-statistic: 42.39 on 13 and 658 DF,  p-value: < 2.2e-16
```

### Model 3 (MD+FX dataset with energy terms only)

```
## 
## Call:
## lm(formula = f, data = dataset)
## 
## Residuals:
##     Min      1Q  Median      3Q     Max 
## -3.9884 -0.5961 -0.2227  0.4390  5.1328 
## 
## Coefficients:
##                 Estimate Std. Error t value Pr(>|t|)    
## (Intercept)      0.79436    0.04876  16.291  < 2e-16 ***
## energy_solvh    -0.53823    0.12846  -4.190 3.17e-05 ***
## energy_vdw       1.12583    0.21595   5.213 2.48e-07 ***
## energy_vdwclash  0.35055    0.07467   4.695 3.24e-06 ***
## entropy_mainc   -0.41686    0.11128  -3.746 0.000195 ***
## entropy_sidec    0.56300    0.11519   4.887 1.28e-06 ***
## total            0.27030    0.06580   4.108 4.49e-05 ***
## ---
## Signif. codes:  0 '***' 0.001 '**' 0.01 '*' 0.05 '.' 0.1 ' ' 1
## 
## Residual standard error: 0.9824 on 665 degrees of freedom
## Multiple R-squared:  0.4243, Adjusted R-squared:  0.4191 
## F-statistic: 81.67 on 6 and 665 DF,  p-value: < 2.2e-16
```

### Model 2 (FX dataset with energy terms and biochemical properties)

```
## 
## Call:
## lm(formula = f, data = dataset)
## 
## Residuals:
##     Min      1Q  Median      3Q     Max 
## -2.9155 -0.7830 -0.2253  0.4942  6.6855 
## 
## Coefficients:
##                 Estimate Std. Error t value Pr(>|t|)    
## (Intercept)      1.23137    0.35177   3.501 0.000496 ***
## P                1.00947    0.25341   3.983 7.55e-05 ***
## strE             0.35642    0.35367   1.008 0.313939    
## strG            -0.15406    0.46489  -0.331 0.740458    
## strH            -0.01823    0.34652  -0.053 0.958054    
## strNONE          0.23781    0.35541   0.669 0.503657    
## strS             0.07806    0.38836   0.201 0.840759    
## strT            -0.14930    0.36693  -0.407 0.684221    
## RSA             -0.81982    0.23030  -3.560 0.000398 ***
## energy_vdw       0.96561    0.13642   7.078 3.75e-12 ***
## energy_vdwclash  0.54087    0.05879   9.201  < 2e-16 ***
## entropy_sidec    0.88574    0.15342   5.773 1.20e-08 ***
## sideHbond        0.27867    0.08260   3.374 0.000785 ***
## total           -0.17205    0.04395  -3.915 9.97e-05 ***
## ---
## Signif. codes:  0 '***' 0.001 '**' 0.01 '*' 0.05 '.' 0.1 ' ' 1
## 
## Residual standard error: 1.244 on 658 degrees of freedom
## Multiple R-squared:  0.2335, Adjusted R-squared:  0.2183 
## F-statistic: 15.42 on 13 and 658 DF,  p-value: < 2.2e-16
```

### Model 1 (FX dataset with energy terms only)

```
## 
## Call:
## lm(formula = f, data = datasets[[i]])
## 
## Residuals:
##     Min      1Q  Median      3Q     Max 
## -2.7496 -0.8337 -0.3130  0.5492  6.8873 
## 
## Coefficients:
##                 Estimate Std. Error t value Pr(>|t|)    
## (Intercept)      1.11077    0.06047  18.368  < 2e-16 ***
## energy_vdw       1.04169    0.13730   7.587 1.11e-13 ***
## energy_vdwclash  0.57383    0.05940   9.661  < 2e-16 ***
## entropy_sidec    0.87501    0.15603   5.608 3.00e-08 ***
## sideHbond        0.31656    0.08402   3.768 0.000179 ***
## total           -0.15691    0.04422  -3.549 0.000415 ***
## ---
## Signif. codes:  0 '***' 0.001 '**' 0.01 '*' 0.05 '.' 0.1 ' ' 1
## 
## Residual standard error: 1.28 on 666 degrees of freedom
## Multiple R-squared:  0.1782, Adjusted R-squared:  0.172 
## F-statistic: 28.87 on 5 and 666 DF,  p-value: < 2.2e-16
```

## Model details: Bind

### Model 5 (MD+FX dataset with all predictors)

```
## 
## Call:
## lm(formula = f, data = datasets[[i]])
## 
## Residuals:
##     Min      1Q  Median      3Q     Max 
## -4.1833 -0.7628 -0.2004  0.6327  4.4735 
## 
## Coefficients:
##                      Estimate Std. Error t value Pr(>|t|)    
## (Intercept)           0.83480    0.53903   1.549 0.122086    
## energy_vdwclash       0.41859    0.09988   4.191 3.28e-05 ***
## backbone_vdwclash_sd  6.68903    1.86318   3.590 0.000363 ***
## energy_vdwclash_sd   -1.06434    0.30689  -3.468 0.000569 ***
## entropy_sidec_sd     -1.21387    0.36372  -3.337 0.000908 ***
## total_sd              1.31652    0.28486   4.622 4.85e-06 ***
## strE                  0.24080    0.53816   0.447 0.654738    
## strG                 -0.61748    0.78859  -0.783 0.433983    
## strH                  0.11572    0.53748   0.215 0.829613    
## strNONE              -0.23644    0.53509  -0.442 0.658775    
## strS                  0.33596    0.55779   0.602 0.547243    
## strT                  0.25023    0.55073   0.454 0.649764    
## RSA                  -1.44965    0.28077  -5.163 3.51e-07 ***
## ---
## Signif. codes:  0 '***' 0.001 '**' 0.01 '*' 0.05 '.' 0.1 ' ' 1
## 
## Residual standard error: 1.172 on 502 degrees of freedom
## Multiple R-squared:  0.5764, Adjusted R-squared:  0.5663 
## F-statistic: 56.93 on 12 and 502 DF,  p-value: < 2.2e-16
```

### Model 4 (MD+FX dataset with energy terms and biochemical properties)

```
## 
## Call:
## lm(formula = f, data = dataset)
## 
## Residuals:
##     Min      1Q  Median      3Q     Max 
## -3.6758 -0.7261 -0.2102  0.5850  4.4705 
## 
## Coefficients:
##                 Estimate Std. Error t value Pr(>|t|)    
## (Intercept)      1.02696    0.53594   1.916  0.05591 .  
## energy_vdw       0.66171    0.14001   4.726 2.98e-06 ***
## energy_vdwclash  0.56079    0.03073  18.250  < 2e-16 ***
## entropy_sidec    0.40532    0.14998   2.702  0.00712 ** 
## P                0.77139    0.25903   2.978  0.00304 ** 
## charge           0.23269    0.08968   2.595  0.00975 ** 
## strE             0.32473    0.54673   0.594  0.55281    
## strG            -0.47358    0.79723  -0.594  0.55276    
## strH             0.22786    0.54816   0.416  0.67782    
## strNONE         -0.16078    0.54272  -0.296  0.76716    
## strS             0.41552    0.56900   0.730  0.46557    
## strT             0.33714    0.55830   0.604  0.54621    
## RSA             -1.93759    0.30466  -6.360 4.55e-10 ***
## ---
## Signif. codes:  0 '***' 0.001 '**' 0.01 '*' 0.05 '.' 0.1 ' ' 1
## 
## Residual standard error: 1.184 on 502 degrees of freedom
## Multiple R-squared:  0.5684, Adjusted R-squared:  0.558 
## F-statistic: 55.08 on 12 and 502 DF,  p-value: < 2.2e-16
```

### Model 3 (MD+FX dataset with energy terms only)

```
## 
## Call:
## lm(formula = f, data = dataset)
## 
## Residuals:
##     Min      1Q  Median      3Q     Max 
## -3.8306 -0.7865 -0.3563  0.5602  4.8711 
## 
## Coefficients:
##                 Estimate Std. Error t value Pr(>|t|)    
## (Intercept)      0.89318    0.06742  13.248  < 2e-16 ***
## energy_vdw       0.70265    0.13544   5.188 3.07e-07 ***
## energy_vdwclash  0.61049    0.02686  22.732  < 2e-16 ***
## entropy_sidec    0.69887    0.16491   4.238 2.68e-05 ***
## sideHbond        0.34815    0.11477   3.034  0.00254 ** 
## ---
## Signif. codes:  0 '***' 0.001 '**' 0.01 '*' 0.05 '.' 0.1 ' ' 1
## 
## Residual standard error: 1.234 on 510 degrees of freedom
## Multiple R-squared:  0.5235, Adjusted R-squared:  0.5198 
## F-statistic: 140.1 on 4 and 510 DF,  p-value: < 2.2e-16
```

### Model 2 (FX dataset with energy terms and biochemical properties)

```
## 
## Call:
## lm(formula = f, data = dataset)
## 
## Residuals:
##     Min      1Q  Median      3Q     Max 
## -3.9231 -0.8088 -0.1922  0.6766  6.4326 
## 
## Coefficients:
##                  Estimate Std. Error t value Pr(>|t|)    
## (Intercept)      0.943374   0.593488   1.590 0.112570    
## P                1.051007   0.284008   3.701 0.000239 ***
## charge           0.349745   0.099823   3.504 0.000500 ***
## strE             0.443849   0.601112   0.738 0.460631    
## strG            -0.476861   0.873829  -0.546 0.585505    
## strH             0.522033   0.606327   0.861 0.389664    
## strNONE          0.005582   0.600648   0.009 0.992589    
## strS             0.394712   0.623907   0.633 0.527254    
## strT             0.313897   0.614136   0.511 0.609492    
## RSA             -2.103408   0.339777  -6.191 1.25e-09 ***
## electro         -0.384853   0.108263  -3.555 0.000414 ***
## energy_solvh    -0.664672   0.145898  -4.556 6.57e-06 ***
## energy_vdw       1.740422   0.236742   7.352 8.06e-13 ***
## energy_vdwclash  0.654139   0.028039  23.330  < 2e-16 ***
## entropy_sidec    0.412430   0.142703   2.890 0.004018 ** 
## ---
## Signif. codes:  0 '***' 0.001 '**' 0.01 '*' 0.05 '.' 0.1 ' ' 1
## 
## Residual standard error: 1.296 on 500 degrees of freedom
## Multiple R-squared:  0.6674, Adjusted R-squared:  0.6581 
## F-statistic: 71.67 on 14 and 500 DF,  p-value: < 2.2e-16
```

### Model 1 (FX dataset with energy terms only)

```
## 
## Call:
## lm(formula = f, data = datasets[[i]])
## 
## Residuals:
##     Min      1Q  Median      3Q     Max 
## -3.7395 -0.8448 -0.3145  0.5886  6.1593 
## 
## Coefficients:
##                   Estimate Std. Error t value Pr(>|t|)    
## (Intercept)        1.02352    0.06813  15.023  < 2e-16 ***
## energy_ionisation  3.79653    1.49121   2.546   0.0112 *  
## energy_solvh      -0.77734    0.14303  -5.435 8.52e-08 ***
## energy_vdw         2.07094    0.23270   8.900  < 2e-16 ***
## energy_vdwclash    0.68420    0.02516  27.189  < 2e-16 ***
## entropy_sidec      0.66242    0.14098   4.699 3.37e-06 ***
## ---
## Signif. codes:  0 '***' 0.001 '**' 0.01 '*' 0.05 '.' 0.1 ' ' 1
## 
## Residual standard error: 1.358 on 509 degrees of freedom
## Multiple R-squared:  0.6284, Adjusted R-squared:  0.6248 
## F-statistic: 172.2 on 5 and 509 DF,  p-value: < 2.2e-16
```
